# Cow dung: a potential biomass substrate for the production of detergent-stable dehairing protease by alkaliphilic *Bacillus subtilis* strain VV

**DOI:** 10.1186/2193-1801-1-76

**Published:** 2012-12-22

**Authors:** Ponnuswamy Vijayaraghavan, Aija Vijayan, Arumugaperumal Arun, John Kennady Jenisha, Samuel Gnana Prakash Vincent

**Affiliations:** 1 International Centre for Nanobiotechnology, Centre for Marine Science and Technology, Manonmaniam Sundaranar University, Rajakkamangalam–629 502, Kanyakumari District, Tamil Nadu India; 2 Centre for Marine Science and Technology, Manonmaniam Sundaranar University, Rajakkamangalam–629 502, Kanyakumari District, Tamil Nadu India; 3 Department of Biotechnology, Kalasalingam University, Virudhunagar, Srivilliputtur, 626 126 Tamilnadu India

**Keywords:** Cow dung, Solid-state fermentation, *Bacillus subtilis* strain VV, Alkaline protease, Detergent-stable

## Abstract

Cow dung, a cheap and easily available source of energy, was used as the substrate for the production of alkaline protease by solid-state fermentation using the *Bacillus subtilis* strain VV. In order to achieve the maximum yield of this enzyme, the following optimum process parameters are needed: fermentation period (72 h), pH (10.0), moisture content (140%), inoculum (25%), temperature (30–40°C), carbon source (2% (w/w) maltose) and nitrogen source (1% (w/w) urea). The protease was stable over a broad temperature range (30–50°C) and pH (8.0-10.0), with maximum activity at 50°C and pH 10.0. Among the divalent ions tested, Ca^2+^ (0.01 M) increased enzyme activity. The purified protease, after being subjected to sodium dodecyl sulphate-polyacrylamide gel electrophoresis, was found to have a molecular mass of 38.5 kDa. The enzyme was solvent-and surfactant-stable and showed activity even after 24 h incubation along with various commercially available detergents. This enzyme possessed dehairing properties for animal hide after 16 h of incubation at room temperature. From these results it is evident that cow dung is a potential substrate for the production of a detergent-stable, dehairing protease by *B. subtilis*. This enzyme has a lot of potential applications in the detergent and leather-processing industries.

## Background

Proteases constitute one of the commercially important groups of extra-cellular microbial enzymes and are widely used in the detergent, food, pharmaceutical, chemical and leather industries (Scheuer 1990 ). These enzymes account for 40% of the total enzyme sales worldwide and this trend is expected to increase in the near future. This has led to increasing attention towards the exploitation of potent microbial strains for the production of alkaline proteases from an industrial point of view (Ellaiah et al. 2002 ). Although a wide range of microorganisms are known to produce proteases, a large proportion of the commercially available form of these enzymes is derived from *Bacillus* strains because of their ability to secrete large amounts of alkaline proteases having significant proteolytic activity and stability at considerably higher pH and temperatures ( Jacobs 1995 ; Yang et al. 2000 ).

The leather processing industry contributes significantly to the country’s economic development. Waste from the leather industry leads to environmental pollution. Alkaline proteases have dehairing properties and can be used in the leather processing industry. Conventional methods in leather processing involve the use of hydrogen sulphide and other chemicals which are pollutants. Thus, for environmental reasons, the enzymatic dehairing process has more advantages over the chemical dehairing process ( Andersen 1998 ). Proteases are used during the soaking, dehairing and bating states of preparing skins and hides. Pancreatic proteases are used in the bating process and the use of microbial alkaline proteases are popular (Varela et al. 1997 ). Alkaline proteases swell hair roots and attack hair follicle proteins, resulting in the easy removal of hair. These enzymes have been widely studied and their production from *Bacillus* sp. has gained momentum; moreover, the high activity and stability of these enzymes at various temperature and pH ranges have also attracted the attention of researchers. Dehairing proteases have been characterized from various *Bacillus* sp., e.g. *B. subtilis* 11QDB32 (Varela et al. 1997 ), *B. amyloliquefaciens* (George et al. 1995 ), *B. subtilis* K2 (Hameed et al. 1996 ; Hameed et al. 1999 ) and *B. circulans* (Subba Rao et al. 2009 ).

Proteases are generally produced using submerged fermentation not only due to its apparent advantages in consistent enzyme production but also for its cost- for medium components. From an industrial point of view, it is estimated that around 30-40% of the production cost of industrial enzymes can be attributed to the cost of the growth medium (Joo et al. 2003 ). Solid-state fermentation (SSF) has gained importance in the production of microbial enzymes owing to several economic advantages over submerged fermentation. The advantages of SSF include lower manufacturing costs with increased production, less pre-processing energy and effluent generation, along with easy process management and better product recovery (Prakasham et al. 2006 ; Oliveira et al. 2006 ). There are several reports describing the use of agro-industrial residues for the production of alkaline protease (e.g. pigeon pea and *Bacillus* sp. JB-99 (Johnvesly et al. 2002 ); green gram husk and *Bacillus* sp. (Prakasham et al. 2006 ); *Imperata cylindrical* grass and potato peel and *Bacillus subtilis* (Mukherjee et al. 2008 ). Apart from these agro-industrial residues, increased attention has been paid in recent times to utilize other waste substances, e.g. feather meal, corn steep liquor (De Azeredo et al. 2006 ) and proteinaceous tannery solid waste, for the production of alkaline proteases (Ravindran et al. 2011 ). Even though cow dung is considered a waste, it contains essential nutrients (Misra et al. 2003 ); these include carbon, nitrogen, phosphorus, potassium, calcium, magnesium, sulphur, manganese, copper, zinc, chloride, boron, iron and molybdenum.

Most of the commercial proteases producing organisms are *Bacillus* sp. (Abo-Aba et al. 2006 ). The potential of cow dung as a biomass substrate for the production of alkaline protease by using *Bacillus* sp. has not yet been completely exploited. The main objective of the present study was the production of alkaline proteases by *Bacillus subtilis* utilizing cow dung as an energy source, and determination of the optimum conditions necessary for the production of these enzymes.

## Results and discussion

### Isolation and identification of a best alkaline protease-producing organism

Of the tested isolates, five were found to have the ability to produce alkaline protease. Among the positive isolates, the organism which produced a larger halo zone in response to the colony diameter was selected. The selected isolate was identified as *Bacillus* on the basis of various microscopic and biochemical investigations. The organism was a Gram-positive rod, spore-producing, VP-, catalase- and gelatin-positive. It fermented glucose, lactose and sucrose. It reacted negatively in the indole, methyl red, citrate, oxidase, starch and nitrate reduction test. All these results suggest that it belongs to the genus *Bacillus*. Moreover, the organism was confirmed by its 16S rRNA gene sequence and identified as *Bacillus subtilis* strain VV. The 1071-bp sequence was submitted to GenBank (accession number: JQ 425476).

### Evaluation of cow dung as a cheap substrate for alkaline protease production

This study has indicated that cow dung can be used as a potential substrate for alkaline protease production. Enzyme production by the *B. subtilis* strain VV was to the tune of 4030 ± 128 U/g solid substrate (cow dung) after 72 h of incubation at 37°C. The selection of a cheap substrate in SSF for the production of any metabolites is an important factor from an industrial point of view. Apart from the cost, the availability of the substrate is a critical factor. An ideal substrate is one which is available in large quantities and throughout the year too. Although many cheap agro-industrial residues were evaluated (Prakasham et al. 2006 ; Johnvesly et al. 2002 ; Gessesse 1997 ) for the production of alkaline proteases, the availability of these substrates is seasonal. Apart from agro-industrial wastes, more attention has been paid to the evaluation of solid wastes for the production of alkaline proteases (Ravindran et al. 2011 ; Ganesh Kumar et al. 2008 ). Waste water from the manufacture of shochu was also tried (Morimura et al. 1994 ) for production of proteases. In spite of evaluating these substrates, the search for a novel substrate continues. Recently, we used cow dung as a substrate for the production of a halo-tolerant alkaline protease using a alkalophilic isolate, *Halomonas* sp. PV1 ( Vijayaraghavan and Vincent 2012 ). Of all the alkalophilic microorganisms that have been screened for use in various industrial applications, members of the genus *Bacillus* were found to be predominant and a prolific source of alkaline proteases ( Kumar and Takagi 1999 ). Reports on SSF of cow dung for the production of alkaline protease using *Bacillus* sp. are limited or perhaps not available. Hence, the present investigation aimed to exploit cow dung that is cheap and globally available for alkaline protease production by *Bacillus subtilis*. The protein content of the cow dung medium was evaluated before and after fermentation. The cow dung possessed 80 ± 12 mg protein/g solid substrate, and the organism utilized 40 ± 4.5% of the protein content for the growth and synthesis of protease.

### Effect of fermentation period and pH on alkaline protease production

**Figure 1:**
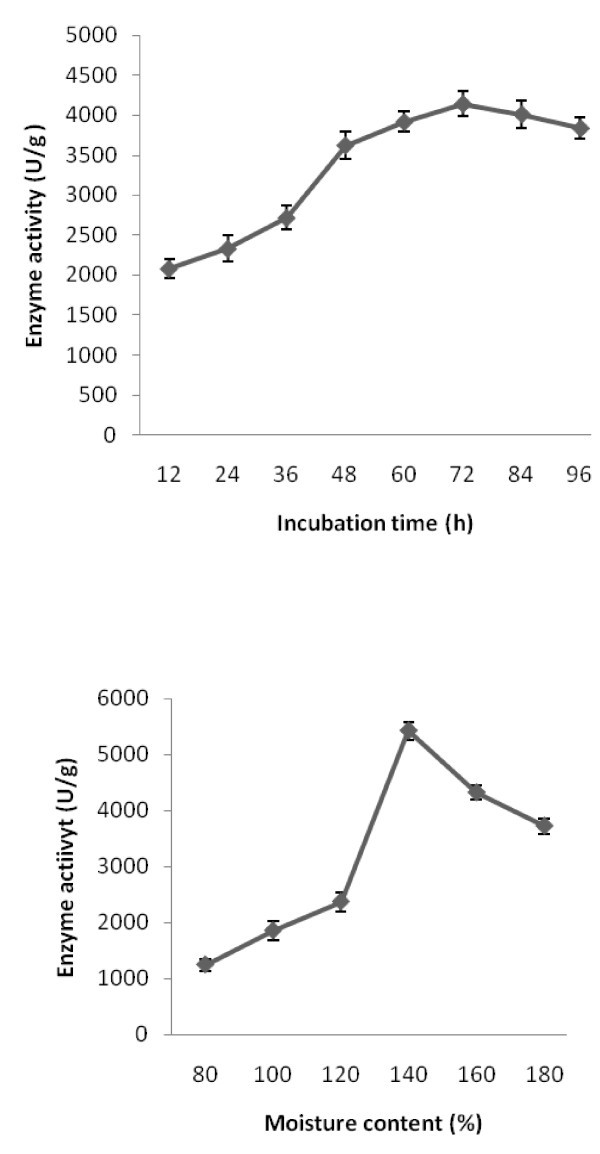
(**a**) **Effect of fermentation period on enzyme production.** The organism was grown in the cow dung substrate and incubated at 37°C for 72 h. *Error bar* standard deviation. (**b**) Influence of initial moisture content on protease production by *Bacillus subtilis* strain VV on cow-dung substrate. *Error bar* standard deviation.

To evaluate the effect of fermentation period on protease production, the fermentation experiment was carried out for a period of 96 h. Results of this study showed that protease production increased with incubation time and was positively correlated (r = 0.842). Maximum alkaline protease production was achieved after 72 h of fermentation (4142 ± 172 U/g substrate) at 37°C (Figure 1a). The incubation time is governed by the characteristics of the culture and is also based on the growth rate and enzyme production. Similar findings have been reported with other *Bacillus* sp. (Ravindran et al. 2011 ). The reduction in enzyme yield after the optimum period was probably due to the depletion of nutrients available to the microorganisms. Here, enzyme production gradually decreased after 72 h. The effect of pH on enzyme production was studied by culturing the organism at various pH levels (6.0-11.0). Enzyme production was 1502 ± 120, 2261 ± 142, 2945 ± 110 and 2291 ± 153 U/g substrate at pH 6.0, 7.0, 8.0 and 9.0, respectively. Enzyme synthesis increased with increase in medium pH (r = 0.839), and maximum enzyme production was achieved at pH 10.0 (4322 ± 148 U/g substrate). This trend clearly implies that this protease producer is alkaliphilic in nature. Enzyme production decreased at pH 11 (3229 ± 129 U/g substrate). At higher pH level (11), protease production decreased as the metabolic action of the bacterium may be suppressed. This result was in accordance with the observations made with other alkaliphilic protease-secreting *Bacillus* sp. ( Uyar and Baysal 2004 ). Alkaline protease production by microbial strains strongly depends on extracellular pH because culture pH strongly influences many enzymatic processes and transport of various components across the cell membranes, which in turn support cell growth and product production (Ellaiah et al. 2002 ).

### Influence of moisture content and inoculum on alkaline protease production

The maximum enzyme production was observed with 140% moisture content (5424 ± 116 U/g substrate). The moisture content positively regulated enzyme production (r = 0.763). The enzyme production decreased thereafter and it was 3722 ± 102 U/g substrate at 180% moisture (Figure 1b). This could be attributed to low microbial growth and anchoring on the surface of the solid medium at higher moisture content. Among the several factors that are important for microbial growth and enzyme production under SSF, moisture content is a critical factor (Pandey et al. 2000 ; Nigam and Singh 1994 ). Cow dung has a high moisture-holding capacity. This could be the reason why the fermentation medium remained loose with higher moisture content and no free water. Even at a higher moisture level (180%), the yield had not decreased much, revealing that this substrate supports the growth of the organism and production of proteases. There was a significant increase in alkaline protease production with an increase in inoculum size and the correlation coefficient (r) was 0.931. Enzyme production was 609 ± 42, 2636 ± 189, 3879 ± 201, 4227 ± 146, 5626 ± 197 and 5094 ± 132 U/g substrate at in inoculum sizes of 5%, 10%, 15%, 20%, 25% and 30%, respectively. Increase of the inoculum level after 25% adversely affected enzyme production. This result was in accordance the results observed with other *Bacillus* sp. (Rajkumar et al. 2011 ).

### Effect of temperature

The effect of temperature on enzyme production was studied by culturing the organism at various temperatures (10–50°C) and the enzyme production was not significantly increased. Enzyme production was 103 ± 27, 2527 ± 91, 3281 ± 127, 4617 ± 101, 5630 ± 162 U/g substrate at temperatures of 10, 15, 20, 25 and 30°C, respectively. The optimum temperature for maximum protease production of 5842 ± 108 U/g substrate in SSF was recorded as 35°C. Incubation of temperatures below 35°C and above 40°C greatly reduced enzyme production. Enzyme production was recorded as 4015 ± 87, 2308 ± 133 and 1134 ± 104 U/g solid substrate at 40, 45 and 50°C, respectively. Incubation temperature is one of the most critical parameters that have to be controlled in the bioprocess as culture temperature influences protease production by microorganism (Ghorbel et al. 2003 ).

### Evaluation of supplementation of carbon and nitrogen sources

The solid medium was supplemented with several carbon sources such as glucose, lactose, trehalose, maltose, xylose and starch at 1% (w/w) level. Among these, the addition of maltose and starch supported maximum production of protease with 5482 ± 118 U/g, and 4360 ± 127 U/g solid substrate respectively. Enzyme production was 2768 ± 82, 4076 ± 103, 3642 ± 121 and 4074 ± 167 U/g substrate when glucose, lactose, xylose and trehalose were added, respectively. When different concentrations of maltose were added, maltose at 2% supported the maximum production with 5629 ± 120 U/g substrate which was statistically significant (r = 0.737). In SSF, addition of carbon sources increases enzyme production. However, addition of the carbon source in the cow dung substrate increased the total enzyme yield by a mere 18%. This clearly implies that cow dung contains more or less enough energy sources for the growth of microorganisms and protease production. The addition of maltose and starch enhanced protease production, with an increase of 30% and 5%, respectively. These results are in accordance with those of another study in which different sugars were supplemented (Ellaiah et al. 2002 ).

**Figure 2:**
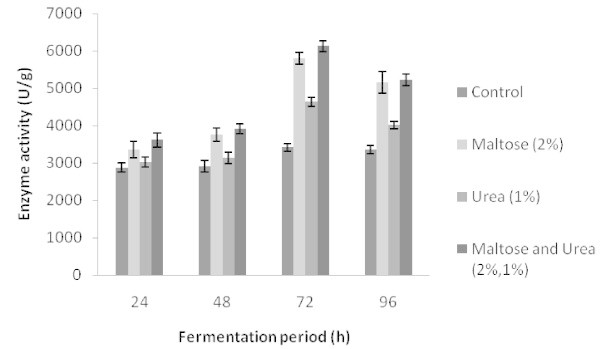
**Effect of maltose, urea, and their combination influence on alkaline protease production by*Bacillus subtilis*strain VV under solid-state fermentation with cow dung.***Error bar* standard deviation.

Among nitrogen sources, addition of urea supported maximum protease production (5412 ± 142 U/g substrate). Addition of other sources such as gelatin (3827 ± 161 U/g), peptone (3485 ± 128 U/g), yeast extract (3587 ± 153 U/g) and casein (4830 ± 134 U/g) also supported protease production. Ammonium chloride repressed protease production (2787 ± 64 U/g). When different concentrations of urea were added, urea at 1% supported maximum protease production with 5489 ± 142 U/g. Enzyme production was not significantly increased in other concentrations and was not statistically significant. Results presented in Figure 2 show the influence of adding maltose, urea and their combinations on alkaline protease production under SSF at varying incubation times.

### Purification of the protease and SDS-PAGE

**Figure 3:**
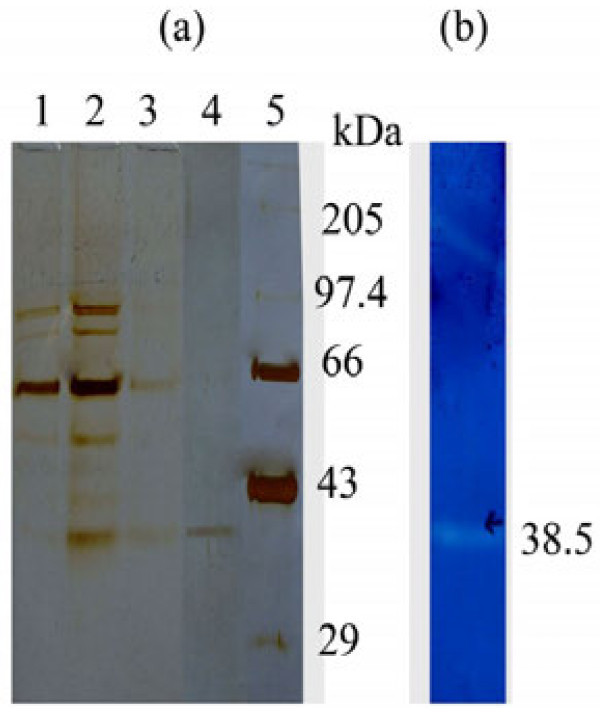
(**a**) **SDS-PAGE analysis of the purified protease.** Lane 1. crude enzyme. Lane 2. ammonium sulphate precipitated sample (upto 40% saturation). Lane 3. ammonium sulphate precipitated sample (80% saturation). Lane 4. purified protease by sephadex G-75. Lane 5. molecular mass marker: 205-myosin muscle rabbit 97.4-phosphorylase b: 66-bovine serum albumin: 43-ovalbumin: 29-carbonic anhydrase. (**b**) zymography of purified protease.

The alkaline protease of the crude extract was purified for homogeneity by a two-step procedure: ammonium sulphate precipitation and Sephadex G-75 gel filtration. In crude extract the specific activity was 16.11 U/mg protein, with an yield of 100%. The alkaline protease was purified 2.45 fold with ammonium sulphate precipitation and further chromatographic separation in Sephadex G-75 column to obtain an 18.37% yield. The specific activity of the purified enzyme was 152.61 U/mg protein. A typical purification experiment is summarized in Table 1. In the SDS-PAGE, the purified enzyme migrated as a single band with an apparent molecular weight of 38.5 kDa (Figure 3a); this was also the case with the zymography analysis (Figure 3b). These results are in accordance with literature reports where molecular masses of most proteases derived from *Bacillus* sp. are less than 50 kDa (Sousa et al. 2007 ).

**Table 1:** **Purification summary of extracellular alkaline protease from*Bacillus subtilis*strain VV**

Purification Step	Total activity (U)	Total protein (mg)	Specific activity (U/mg)	Yield (%)	Purification (fold)
Crude enzyme	16200	1005	16.11	100	1
Ammonium sulphate	9740	246	39.59	60.12	2.45
Sephadex G-75	2976	19.5	152.61	18.37	9.47

### Effect of temperature and pH on the activity of alkaline protease

Enzyme activity was increased with increase in temperature and was statistically significant (p < 0.05). At around 50°C, the maximum protease activity (5922 ± 182 U/g) was determined and it declined at higher temperatures. The enzyme activity was 3062 ± 123, 4432 ± 138, 3802 ± 107, and 994 ± 42 U/g at 30, 40, 60 and 70°C, respectively. Enzyme activity was not detected at 80°C. This might be due to the total denaturation of the enzyme. This protease could be classified as thermostable, because of its optimal activity at 50°C. This result was in accordance with the results obtained with other *Bacillus* sp. (Rajkumar et al. 2011 ). When analyzed for thermal stability, the protease was found to be stable for 40 min at 50°C (42 ± 3.8% activity), which decreased to 2 ± 0.13% after 120 min of denaturation at this temperature. The effect of pH on enzyme activity was evaluated in the pH range, 6.0 to 11.0 and optimal activity occurred in the alkaline range (pH 9.0-11.0). The enzyme produced by the *Bacillus subtilis* strain VV revealed robustness towards alkaline pH. Enzyme activity was found to be the high at higher pH (10.0) (7464 ± 169 U/g material) and was statistically significant (p < 0.05). The enzyme activity was 1156 ± 72, 3548 ± 112, 3631 ± 147, 4140 ± 118 and 4744 ± 152 for the pH 6.0, 7.0, 8.0, 9.0 and 11.0, respectively. This result was in accordance observations made with other *Bacillus* sp. (Ghorbel et al. 2003 ; Arulmani et al. 2007 ). The protease was stable over a pH range of 8.0 to 11.0, and was highly stable at pH 9.0 (100% relative activity). It lost approximately 15.7 ± 1.8% and 33.7 ± 2.6% activity at pH 10.0 and 11.0, respectively. However, more than 61 ± 4.1% of the enzyme activity was lost when the pH fell below 6.0. Similar pattern of pH stability of protease produced by *Bacillus circulans* was described earlier (Towatana et al. 1999 ).

### Effect of ions on enzyme activity

The effect of various divalent ions (0.01 M) on the activity of the enzyme was evaluated. Of the various divalent ions added, Ca^2+^ ions were found to increase enzyme activity (108 ± 4.9%). This result was in accordance with results observed with other *Bacillus* sp. in a similar study (Towatana et al. 1999 ). Ions like Cu^2+^ (64 ± 2.8%), Fe^2+^ (73 ± 5.3%), Hg^2+^ (13.2 ± 1.6%) and Zn^2+^ (61.9 ± 3.4%) strongly inhibited enzyme activity. Enzyme activity was slightly affected by Mg^2+^ and Mn^2+^ ions. The characteristic features of the enzyme produced by *B. subtilis* in this study were similar to that produced by *Bacillus circulans* (Subba Rao et al. 2009 ).

### Effect of organic solvent, surfactants and detergents on the stability of protease

The enzyme was stable towards all tested organic solvent (1%) for 1 h at room temperature. Among the organic solvents tested, methanol and acetonitrile showed better stability (Table 2) but these were not significantly increased. The alkaline protease was stable towards the non-ionic surfactants like SDS, tween-20, tween-80 and triton X-100 and the enzyme activity was 104 ± 2.1%, 168.2 ± 7.8%, 111.5 ± 9.8% and 141.3 ± 21%, respectively. These results were statistically significant (p < 0.05). This finding gains significance because modern bleach-based detergent formulations are mainly composed of SDS. This result is in accordance with the observation made with *Bacillus clausii* (Joo et al. 2003 ). This enzyme showed significant stability (p < 0.05) in the presence of commercially available detergent such as Sunlight and Ujala after 1 h of incubation. This alkaline protease was evaluated for its possible applications in detergent formulation as it showed stability after 1 h and 24 h incubation with these detergents and the results are presented in Table 2. A similar result was reported with *Bacillus circulans* (Subba Rao et al. 2009 ). The enzymatic properties of the protease suggest its suitability as an addition to detergent formulations.

**Table 2:** **Effect of solvent and detergents on protease activity from*Bacillus subtilis*strain VV**

Effect of solvent		Effect of detergents		
Solvent (1%)	Residual activity (%)	Detergent (1%)	Relative activity (%)	
			after 1 h	after 24 h
n-Butanol	66.3 ± 4	Sun light	113 ± 7	50 ± 4
Toluene	77.2 ± 4	mr. White	84 ± 5	14 ± 1
Methanol	102.2 ± 3	Henko	99 ± 4	30 ± 5
Acetone	80.5 ± 3	Ujala	110 ± 5	19 ± 3
Acetonitrile	99.2 ± 8	Tide+	85 ± 6	81 ± 5
Benzene	82.6 ± 6	Surf excel	93 ± 5	31 ± 4
Ethanol	92.5 ± 6	Aircel	13 ± 2	0

The protease was pre-incubated with solvent and surfactants at pre-determined time and the remaining activity was measured using the standard protease assay. Residual and relative enzyme activity was determined as percentage of control with no additions.

### Dehairing of skin

**Figure 4:**
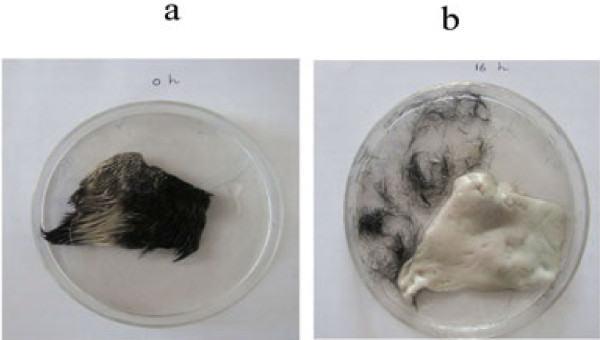
**De-hairing of goat hide at room temperature (30°C) after 16 h of incubation (pH 10.0).** (**a**) Control (0 h); (**b**) Goat hide after 16 h incubation.

In the present study, 4.0 mg enzyme solution effectively removed hair from the goat skin after 16 h of incubation at room temperature (30°C) (Figure 4). This enzyme has non-keratinolytic and non-collagenolytic in nature. Several microbial proteases were evaluated for their dehairing property (Sivasubramanian et al. 2008 ; Aravindan et al. 2007 ) and it was noticed that only those enzymes with stability under alkaline conditions especially between 9.0 and 11.0 are important. *Bacillus subtilis* proteases had many advantages when compared with proteases from other *Bacillus* sp. because of its stability at this range. There is not much published literature concerning enzymatic dehairing process. It is gaining importance as an alternative chemical process and is significant in the reduction of toxicity in addition to the improvement of the texture of leather (Sivsubramanian et al. 2008 ). Alkaline proteases derived from *B. circulans, B. cereus* and *B. subtilis* dehaired the goat skin in 18, 21 and 12 h, respectively (Subba Rao et al. 2009 ; Sivasubramanian et al. 2008 ; Nilegaonkar et al. 2007 ). Based on this fact, alkaline protease derived from *B. subtilis* strain VV can find great use in the leather processing industry.

## Methods

### Isolation of proteolytic microorganisms

Eleven alkaliphilic bacteria were isolated from soil sediments from a small river (Pantivaikal Odai, Pampanvilai, Nagercoil, Kanyakumari District, India). The soil sample collected was found to be highly alkaline due to the extensive use of soap and detergents for cleaning clothes in the river by the local population. All of the isolates were cultured on skimmed milk agar plates (in g l^-1^: [peptone-5; yeast extract-5; KH_2_PO_4_-1.0; MgSO_4_-0.2; skimmed milk-10; NaCl-10; agar-15; pH 10.0]). The isolate having the highest ratio of clear zone diameter (mm) to colony diameter (mm) was selected to test their ability to produce alkaline protease.

### Identification of the potential isolate

Bacterial identification was conducted based on morphological and biochemical tests (Mac Faddin 2000 ). Morphological and physiological characteristics of the isolated bacterium were verified by using ‘Bergey’s Manual of Systematic Bacteriology’ ( Jones and Collins 1984 ). The 16S rRNA gene was sequenced after genomic DNA extraction and PCR amplification as described elsewhere (Riffel et al. 2003 ).

### Solid-state fermentation and protease extraction

Cow dung was obtained from a farm house and dried for several days. It was powdered using a mixer grinder, sieved and stored in airtight containers before further use. Five grams of the substrate (cow dung) was taken in a 250-mL Erlenmeyer flask and the moisture content was maintained as 100% using glycine-NaOH buffer (pH 10.0, 0.1 M). The contents were mixed thoroughly and autoclaved at 121°C for 15 min. After cooling the flask to room temperature, it was inoculated with 0.5 mL of 24-h grown (OD_600 nm_ = 0.917) culture broth under sterile conditions. The culture was then incubated for 72 h at 37°C, and after incubation, 50 mL of double distilled water was added to the fermented substrate. This was placed in an orbital shaker at 150 rpm for 30 min for enzyme extraction. After this, the mixture was rapidly filtered using cotton and the cells were further harvested by centrifugation at 10,000 *g* for 20 min. The supernatant was used as the enzyme source for protease assay.

### Determination of protease activity

Alkaline protease activity was determined by standard assay. The reaction mixture contained 5 mL of casein (prepared in 0.05 M of glycine-NaOH buffer, pH 10.0) and an aliquot of 0.1 mL of the enzyme solution, and this mixture was incubated for 30 min at 37°C. The reaction was stopped by adding 5 mL of trichloroacetic acid solution (TCA) (0.11 M) and the mixture was filtered after 30 min. To 2 mL of the filtrate, 5.0 mL of 0.5 M sodium carbonate and 1.0 mL of Folin-Ciocalteu’s phenol reagent were added, and this mixture was kept undisturbed for 30 min at 37°C. The optical density of the solution was read against sample blank at 630 nm. One unit of enzyme activity was defined as the amount of enzyme required to liberate 1 μg of tyrosine per minute under assay conditions ( Chopra and Mathur 1985 ). The total protein content was estimated by Bradford’s method ( Bradford 1976 ).

### Optimization of process parameters for protease production

In the present study, solid-state protease production by the *Bacillus subtilis* strain VV was optimized by varying the physical parameters and nutrient sources. The protease activity was determined in the fermented medium for every 12 h of fermentation up to 96 h in order to determine the fermentation period. To evaluate the effect of temperature on protease production, the substrate inoculated with bacterial culture was incubated at various temperatures (10–50°C). Addition of buffer (0.1 M) was performed so that the pH of the solid medium was varied from pH 6.0 to 11.0. To study the effect of the initial moisture content on protease production, the initial moisture content of the cow dung was adjusted to 60-180% using glycine-NaOH buffer (pH 10.0). To determine the effect of inoculum size on protease production, the inoculum concentration was increased accordingly (5-30%).

In addition to the physical parameters, nutrient parameters were also optimized. This included the effect of carbon sources (1%, w/w) (glucose, lactose, trehalose, maltose, xylose and starch) and nitrogen sources (1%, w/w) (gelatin, ammonium nitrate, peptone, yeast extract, urea, skimmed milk, and casein). The maximum production of protease at various concentrations (0.5-2.5%) of maltose as a carbon source and urea as a nitrogen source was investigated. The effect of the optimum concentration of maltose, urea and their combination on alkaline protease production for 24–96 h was also evaluated. The results reported in this study are averages of triplicate findings.

### Purification of protease

The organism was grown aerobically in an optimized medium for 72 h at 37°C and extracted as described in materials and method section. Samples were centrifuged at 10,000 *g* for 10 min, and the supernatant was used as a crude enzyme preparation. It was precipitated with ammonium sulphate (40-80% saturation) and the enzyme precipitate obtained was centrifuged at 10,000 *g* for 10 min at 4°C. The precipitate obtained from the previous step was re-suspended in 5.0 mL of 0.025 M Tris–HCl buffer and dialysed against the same buffer. The dialysed sample was applied to a Sephadex G-75 gel filtration column (0.6 × 45 cm), and eluted with 0.025 M Tris–HCl buffer at pH 8.0, at a flow rate of 0.5 mL/min. Fractions of 2.0 mL were collected and the optical density of the sample was measured at 280 nm and analysed for proteolytic activity.

### SDS-PAGE and zymography

Sodium dodecyl sulphate-polyacrylamide gel electrophoresis was carried out according to ( Laemmli 1970 ) using 11% crosslinked polyacrylamide gel. Silver staining was performed to visualize protein bands. Zymographic analysis was performed by the enzyme pattern of proteins, obtained by zymogram with 1% casein substrate and detected using coomassie brilliant blue R-250 (Westergaar et al. 1980 ).

### Characterization of protease activity

The effect of pH on the activity of the enzyme was studied by assaying the enzyme activity at different pH values ranging from 5.0 to11.0. To check the stability of the enzyme at various pH, 100 μL of the enzyme solution was mixed with 900 μL of buffer solutions (pH 5.0-11.0) and the mixture was taken to measure the protease activity under standard assay conditions after incubation for 1 h. The effect of temperature on enzyme activity was studied by holding the reactions at various temperatures (30–70°C) using the standard assay method. To evaluate the heat stability of the protease, the sample was denatured at an optimized temperature (50°C) for 0–120 min. To study the effect of ions (0.01 M) on enzyme activity, the sample was pre-incubated with various divalent ions at 37°C for 1 h and the activity evaluated. To examine the effect of solvents, surfactants and detergents on enzyme activity, many agents were added to the enzyme solution at the indicated concentration, allowed to stand for 1 h and 24 h at room temperature and the activity measured. To evaluate the dehairing property of an enzyme, fresh goat-hide was incubated with 4.0 mg enzyme solution (pH 10.0) for up to 16 h at room temperature.

### Statistical analysis

All experiments were performed in triplicate. Data were analyzed by correlation coefficient (r) and Student’s ‘t’ test. A significance level of 0.05 or less was considered statistically significant.

## Conclusions

In conclusion, cow dung was utilized as a substrate for the production of alkaline protease in SSF. Cow dung is a cheaply available bioresource and this substrate is available in almost every country. So, this biomass could be effectively utilized for the production of alkaline protease an industrial scale. Apart from the significance of the substrate, the enzyme secreted by *B. subtilis* strain VV is useful in the detergent and leather-processing industries.
